# A Heterozygous Missense Variant in *MAP2K2* in a Stillborn Romagnola Calf with Skeletal-Cardio-Enteric Dysplasia

**DOI:** 10.3390/ani11071931

**Published:** 2021-06-29

**Authors:** Joana G. P. Jacinto, Irene M. Häfliger, Arcangelo Gentile, Cord Drögemüller

**Affiliations:** 1 Department of Veterinary Medical Sciences, University of Bologna, Ozzano Emilia, 40064 Bologna, Italy; joana.goncalves2@studio.unibo.it (J.G.P.J.); arcangelo.gentile@unibo.it (A.G.); 2 Institute of Genetics, Vetsuisse Faculty, University of Bern, 3012 Bern, Switzerland; irene.haefliger@vetsuisse.unibe.ch

**Keywords:** cattle, cardiac defect, development, congenital malformations, heterotopy, precision medicine, short spine, RASopathy

## Abstract

**Simple Summary:**

Skeletal dysplasias encompass a clinical-, pathological- and genetically heterogeneous group of disorders characterized by abnormal cartilage and/or bone formation, growth, and remodeling. They may belong to the so-called RASopathies, congenital conditions caused by heterozygous variants in genes that encode components of the Ras/mitogen-activated protein kinase (MAPK) cell signaling pathway. Herein, an affected calf of the Italian Romagnola breed was reported showing a skeletal-cardio-enteric dysplasia. We identified a most likely disease-causing mutation in the *MAP2K2* gene by whole-genome sequencing (WGS). The *MAP2K2* gene is known to be related with dominant inherited cardio-facio-cutaneous syndrome in humans, but it was so far unknown to cause a similar disease in domestic animals. We assume that the identified missense variant that was predicted to impair the function of the protein, occurred either within the germline of the dam or post-zygotically in the embryo. Rare lethal diseases such as the skeletal-cardio-enteric dysplasia in livestock are usually not characterized to the molecular level, mainly because of the lack of funds and diagnostic opportunities. Precise WGS-based diagnostics enables the understanding of rare diseases and supports the value of monitoring cattle breeding populations for fatal genetic defects.

**Abstract:**

RASopathies are a group of developmental disorders caused by dominant mutations in genes that encode components of the Ras/mitogen-activated protein kinase (MAPK) cell signaling pathway. The goal of this study was to characterize the pathological phenotype of a Romagnola stillborn calf with skeletal-cardio-enteric dysplasia and to identify a genetic cause by whole-genome sequencing (WGS). The calf showed reduced fetal growth, a short-spine, a long and narrow face, cardiac defects and heterotopy of the spiral colon. Genetic analysis revealed a private heterozygous missense variant in *MAP2K2:*p.Arg179Trp, located in the protein kinase domain in the calf, and not found in more than 4500 control genomes including its sire. The identified variant affecting a conserved residue was predicted to be deleterious and most likely occurred de novo. This represents the first example of a dominant acting, and most likely pathogenic, variant in *MAP2K2* in domestic animals, thereby providing the first *MAP2K2*-related large animal model, especially in respect to the enteric malformation. In addition, this study demonstrates the utility of WGS-based precise diagnostics for understanding sporadic congenital syndromic anomalies in cattle and the general utility of continuous surveillance for rare hereditary defects in cattle.

## 1. Introduction

Genetic skeletal dysplasias encompass a clinical-, pathological-, and genetically heterogeneous group of rare disorders characterized by abnormal cartilage and/or bone formation, growth, and remodeling [1]. In human medicine, 461 different skeletal disorders are classified into 42 subtypes [2]. At the present in humans, pathogenic variants affecting more than 437 different genes have been found to be associated with these disorders [2]. In veterinary medicine, genetic skeletal dysplasias are not classified in such detail. Nevertheless, with the progressively widespread availability of molecular tools for genetic mapping, such as single-nucleotide polymorphism (SNP) arrays, and for mutation analysis, such as short-read based whole-genome sequencing (WGS), the recognition of disease-causing pathogenic variants has drastically improved [3,4]. In fact, the 1000 Bull Genomes Project now encompasses a genetic variation from over 4100 genomes providing a comprehensive database for the imputation of genetic polymorphisms for genomic prediction in all cattle breeds, improving the accuracy of genomic prediction in the identification of causal mutations [5]. The OMIA (Online Mendelian Inheritance of Animals) currently lists 22 skeletal disorders in cattle with a known causal mutation, e.g., recessively inherited mostly breed-specific disorders such as the brachyspina syndrome in Holstein (OMIA 000151-9913) [6] and the paunch calf syndrome in Romagnola [7] and Marchigiana [8] (OMIA 001722-9913), or dominantly inherited disorders such as the bovine achondrogenesis type II (OMIA 001926-9913) [9], or cases of facial dysplasia in the progeny of a single bull (OMIA 002090-9913) [10]. The latter two diseases have been shown to result from de novo mutation events in the paternal germline.

This study aimed to characterize in detail the pathological phenotype of a Romagnola calf with skeletal cardio-enteric dysplasia, and to discover a genetic variant causing the abnormality by WGS.

## 2. Materials and Methods

### 2.1. Pathological Investigation

A stillborn Romagnola male calf was referred to the Department of Veterinary Medical Sciences, University of Bologna for post-mortem examination. The calf resulted from insemination with semen from a purebred Romagnola bull on a Romagnola cow. The pedigree of both parents showed no common ancestor within four generations. The truncal length was measured from the occipital bone to the tuber coxae. Additionally, radiographic images of the spine were obtained before starting necropsy.

### 2.2. DNA Samples

Genomic DNA was isolated from skin and cartilage from the ear of the calf and from the semen of the sire using Promega Maxwell RSC DNA system (Promega, Dübendorf, Switzerland). In addition, genomic DNA samples from EDTA-blood samples of 100 apparently normal Romagnola cattle were extracted with the same methodology and used as controls.

### 2.3. Whole-Genome Sequencing

The genome of the affected calf was sequenced as described before resulting in an average read coverage of approximately 17.4× [11]. Single-nucleotide variants (SNVs) and small indel variants were called subsequently as reported earlier [5], except for the trimming, which was carried out using fastp [12]. Further data processing was carried out according to Häfliger et al., 2020 [13]. The impact of the called sequence variants was evaluated with snpeff v4.3 [14], using NCBI Annotation Release 106 (https://www.ncbi.nlm.nih.gov/genome/annotation_euk/Bos_taurus/106/; accessed on 30 April 2021). In order to search for private variants, we compared the genotypes of the affected calf with 598 bovine genomes of different breeds that have been sequenced in other ongoing studies and are freely available (Table S1) in the European Nucleotide Archive (SAMEA7690195 is the sample accession number of the affected calf; http://www.ebi.ac.uk/en; acceded on 30 April 2021). The occurrence of these variants was then investigated in a global control cohort of 4110 genomes of different breeds (1000 Bull Genomes Project run 8; www.1000bullgenomes.com; acceded on 30 April 2021) [5]. Integrative Genomics Viewer (IGV) [15] software version 2.0 was used to manually look at genomic regions where possible candidate genes map.

### 2.4. Targeted Genotyping

PCRs were carried out using Amplitaq Gold Master Mix (Thermofisher, Rotkreuz, Switzerland). The subsequent bi-directional sequencing of PCR products was carried out after shrimp alkaline phosphatase (Roche, Basel, Switzerland) and exonuclease I (NEB, Axon lab, Baden, Switzerland) incubation using the PCR primers with the ABI BigDye Terminator Sequencing Kit 3.1 (Applied Biosystems, Zug, Switzerland) on an ABI 3730 capillary sequencer (Applied Biosystems). The *MAP2K2* missense variant (NM_001038071.2: g.19923991C>T) was genotyped using the following primers: 5′- GGCTTAACAGAGGATGCCCC-3′ (forward primer) and 5′-CTGGAAAACCTGGAAATCGGG-3′ (reverse primer). The evaluation of the sequence data was carried out with Sequencher 5.1 software (GeneCodes, Ann Arbor, MI, USA).

### 2.5. Evaluation of the Molecular Consequences of Amino Acid Substitutions

PROVEAN [16] and PredictSNP1 [17] were used to predict the biological consequences of the discovered variant on protein. For multispecies sequence alignments the following NCBI proteins accessions were used: NP_001033160.2 (*Bos taurus*), NP_109587.1 (*Homo sapiens*), XP_003318859.1 (*Pan troglodytes*), XP_001118016.2 *(Macaca mulatta*), NP_001041601.1 (*Canis lupus*), NP_075627.2 (*Mus musculus*), NP_579817.1 (*Rattus norvegicus*), NP_990719.1 (*Gallus gallus*), NP_001032468.2 (*Danio rerio*).

### 2.6. Sequence Accessions

Genomic positions in the cow genome refers to the the ARS-UCD1.2 assembly. All references to the bovine *MAP2K2* gene correspond to the NCBI accessions NC_037334.1 (chromosome 7, ARS-UCD1.2), NM_001038071.2 (*MAP2K2* mRNA), and NP_001033160.2 (*MAP2K2* protein). For the *MAP2K2* protein, the Uniprot database (https://www.uniprot.org/) (accessed on 15 March 2021) accession number A0A3S5ZPX3 was used.

## 3. Results

### 3.1. Pathological Phenotype

**Figure 1:**
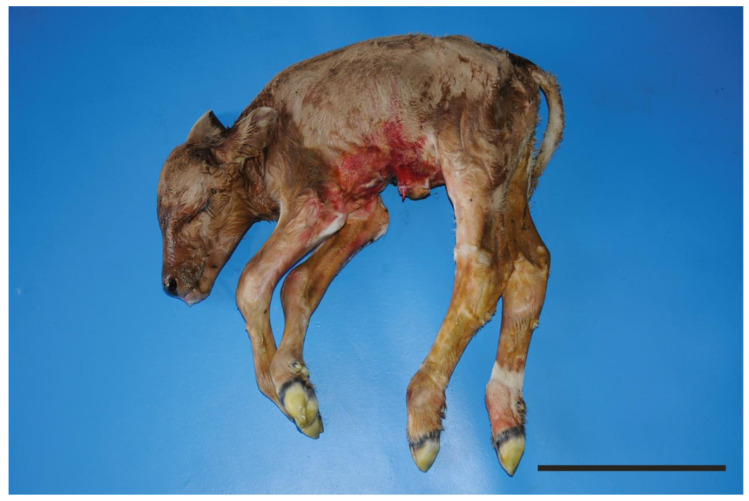
Stillborn Romagnola calf with skeletal-cardio-enteric dysplasia: Strikingly, the length of the spine appeared disproportionately short for the height and legs. Mild kyphosis of the thoraco-lumbar vertebral column is also evident. Bar, 30 cm.

At birth, the calf weighed only 20.3 kg (normal weight of Romagnola calves at birth 40 Kg–mean) and its trunk was disproportionately short for the body size and legs (Figure 1; Figure S1). Shortening of the neck was very pronounced, and it looked as though the head was fixed to the chest. The length of trunk, as measured from the occipital bone to the point of the buttock, was approximately 40 cm. Moreover, it displayed a mild kyphosis at the level of the thoracolumbar region. The limbs were 70 cm long and slender (dolichostenomelia).

Facial deformities were characterized by narrow, longer and laterally deviated splancnocranium. The lower jaw was slightly longer than normal.

Radiological examination of the axial skeleton showed only a reduced size of the vertebral bodies, but no shape abnormalities (Figure S2).

**Figure 2:**
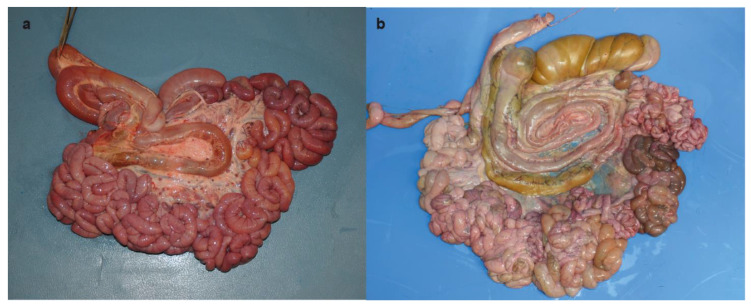
Topography of the spiral loop of the ascending colon. (**a**) Topography of the spiral loop of the ascending colon in the stillborn Romagnola calf with skeletal-cardio-enteric dysplasia. Note the complete displacement of the spiral loop of the ascending colon from the mesojejunum. (**b**) Topography of the spiral loop of the ascending colon in a control. Note the spiral loop of the ascending colon located at the middle of the mesojejunum.

At gross pathology, the examination of the abdominal cavity showed the absence of the omentum and heterotopy of the spiral colon, the latter characterized by a complete detachment and complete displacement of the spiral loop of the ascending colon from the mesojejunum (Figure 2a,b).

The calf also showed an evident 2 cm diameter persistent patent ductus and pathological cardiac abnormalities, including globous shape, enlarged right ventricle and pulmonic stenosis.

Based on these pathological observations, the animal was considered to present a skeletal-cardio-enteric dysplasia.

### 3.2. Genetic Analysis

**Figure 3:**
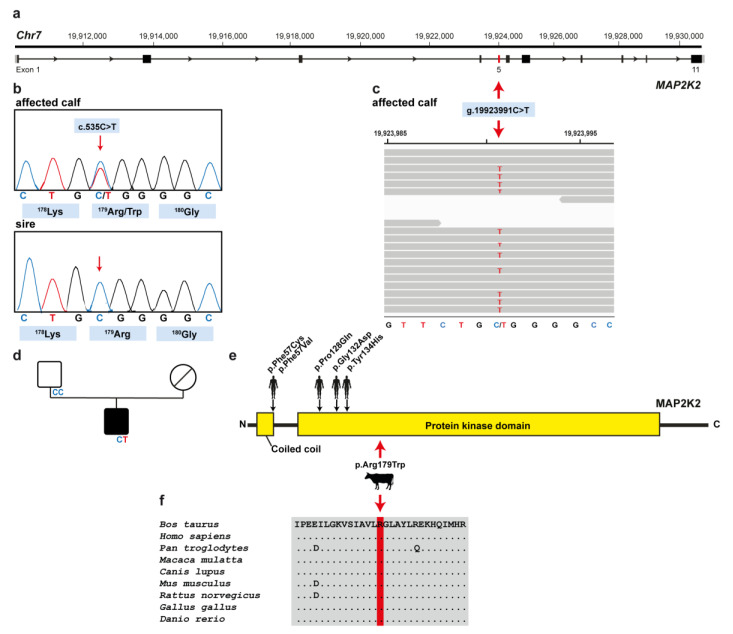
*MAP2K2* missense variant in a Romagnola calf with skeletal-cardio-enteric dysplasia: (**a**) *MAP2K2* gene structure representation of the exact position of the exon 5 variant on the chromosome 7 (red arrow); (**b**) Electropherograms confirming heterozygosity in the affected calf and the absence of the variant in the germline of the sire. (**c**) IGV screenshot presenting the Chr7: g.19923991C>T variant in the calf. (**d**) Pedigree of the case. The affected male calf is represented with full black symbol, while both non-affected parents are represented by full white symbols. Unknown genotype is represented by a symbol with a diagonal line. (**e**) Schematic representation of *MAP2K2* protein and its functional domains and summary of known human *MAP2K2* mutations. The position of the mutation detected in the affected Romagnola calf is indicated by a red arrow, while know human *MAP2K2* mutations associated with cardio-facio-cutaneous syndrome are indicated by black arrows (OMIM601263). (**f**) Multiple sequence alignment of the protein kinase domain of the *MAP2K2* protein around the position of the p.Arg179Trp variant shows complete evolutionary conservation across all species.

Assuming a spontaneous mutation as the most likely cause for this congenital malformation, the whole genome of the affected calf was sequenced. To evaluate the presence of coding protein-changing variants, filtering of WGS for private variants present in the calf and not present in the 598 control genomes was performed. This approach identified 381 heterozygous private protein-changing variants predicted to have moderate or severe alteration on the encoded proteins. These variants were then tested for their presence in a global control cohort of 4110 genomes of various breeds collected in run 8 of the 1000 Bull Genomes Project [5], which revealed 66 protein-changing variants only occurring in heterozygous state in the genome of the affected calf. These 66 variants were subsequently evaluated using IGV software, confirming 63 as true variants (Table S2). Of all the remaining private variants, one occurred in a possible candidate gene for observed phenotype (Figure 3a). The heterozygous variant at chr7:19923991C>T represents a missense variant in exon 5 of the *MAP2K2* gene (NM_001038071.2: c.535C>T; Figure 3c). This variant alters the amino acid of the *MAP2K2* protein at site 179 (NP_001033160.2: p.Arg179Trp) located in the protein kinase domain (Figure 3e). Furthermore, the arginine to tryptophan substitution affects an evolutionary conserved residue (Figure 3f) and has been predicted to be harmful (PROVEAN score −4.708; Predict SNP score 61%). In order to confirm and finally evaluate the *MAP2K2* variant, the corresponding genome region was amplified by PCR and then analyzed by Sanger sequencing in the calf, its sire, and 100 controls of the same breed. Unfortunately, no biological sample of the dam that was slaughtered in-between was available. When analyzing the sequencing data, we found that the calf was indeed heterozygous for the *MAP2K2* variant detected, while the sire and another 100 controls from the Italian Romagnola population were homozygous for the wild-type allele (Figure 3b,c).

In addition, when looking for homozygous variants, filtering revealed two private protein-changing variants present in the genome of the affected calf (Table S2).

## 4. Discussion

Non-infectious syndromic congenital malformations of newborns in cattle occur rarely and are most often not further diagnosed in detail. We have carried out a comprehensive pathological and genetic examination in a Romagnola stillborn calf, revealing a skeletal-cardio-enteric dysplasia. We then investigated the hypothesis of a spontaneous mutation as a possible reason for this congenital phenotype. Using state-of-the-art genetic approaches involving WGS, geneticist have only about a 50:50 chance of quickly identifying variants that are causal for developmental anomalies in humans [18]. So far, similar data is missing for veterinary medicine, mostly due to the lack of resources, although the scientific value for biomedical research is widely accepted [4]. Analysis of the genome sequence of the studied case identified a missense variant in a plausible candidate gene affecting the protein kinase domain of *MAP2K2*. In addition, this variant was only present in the genome of the affected calf, and did not occur in a global control cohort of more than 4500 bovine genomes of different breeds. Therefore, considering the rarity of this coding variant and the function of the *MAP2K2*, it was considered to represent the most likely genetic cause for the observed phenotype. To the best of our knowledge, no pathogenic variant in the *MAP2K2* gene has been reported in domestic species. Therefore, this study represents the first large animal model for a *MAP2K2*-related congenital skeletal disorder in cattle.

Furthermore, the PCR to detect the mutant allele in the sire using DNA extracted from semen showed a homozygous wild-type genotype. Therefore, we could exclude the father as a mosaic ancestor. However, to definitively prove that the identified variant in *MAP2K2* indeed occurred de novo, genotyping of the dam would be needed. Therefore, we speculate that the mutation either arose post-zygotically during fetal development of the affected calf or represents a maternally derived germline mutation.

The RAS/mitogen activated protein kinase (MAPK) cell signaling pathway plays an important role in the regulation of the cell cycle and differentiation [19]. In particular, during embryonic development, it represents one of the main pathways for the transduction of intracellular signals in response to all types of mitogens (e.g., growth factors), which initiates proliferation, survival, and anti-apoptotic programs [20]. Furthermore, the dysregulation of RAS/MAPK-dependent developmental processes has significant pathophysiological consequences [20].

In humans, somatic mutations leading to hyperactivation of the RAS/MAPK signaling cascade may cause cancers [21], whereas germline or de novo mutations in the developing embryo are responsible for several rare genetic conditions, collectively termed RASopathies. These disorders have common phenotypes, such as a short stature, heart defects, facial abnormalities, and cognitive impairments, often associated with abnormal central nervous system development, and include conditions such as neurofibromatosis type 1 (OMIM 162200) [22], Noonan (OMIM 163950) [23], LEOPARD (OMIM 151100) [24], Costello (OMIM 218040) [25], and cardio-facio-cutaneous (CFC; OMIM 115150) [26] syndromes. In humans, RASopathies are a highly heterogenetic group of genetic disorders, being associated to more than 20 causal genes [20]. These genes encode proteins that belong to, or regulate, the RAS/MAPK cell signaling pathway. Their mutations explain the pathophisological mechanisms, such as the abnormal development of various tissues (e.g., cardiac or craniofacial defects), as well as the altered hormonal response and consequent endocrine dysfunctions (e.g., growth hormone insensitivity, and growth retardation) [20]. In particular, individuals affected by CFC show characteristic craniofacial dysmorphic features, short stature, cardiac defects, ectodermal anomalies, and developmental delay [26]. Currently, dominantly inherited mutations in four genes have been associated with CFC syndrome: *BRAF* [27], *MAP2K1* [28], *MAP2K2* [26,28], and *KRAS* [27]. Interestingly, the affected calf presented in this study revealed a dominant mutation in *MAP2K2*, and showed retarded growth, skeletal dimorphisms, including a long and narrow face and shortening of the vertebral column, and cardiac defects such as persistent patent ductus and pulmonary stenosis. These findings resemble the phenotype of human CFC syndrome. However, the animal did not show any alteration on the integumentary system.

Moreover, the calf revealed heterotopy of the spiral colon. In cattle, this anatomic anomaly may originate from the loose attachment of the spiral loops to the mesojejunum, abnormal elongation of the mesocolon, overlapping of adjacent loops, abnormal coiling, and finally, complete separation of the spiral loop of the ascending colon from the mesojejunum [29]. This condition can predispose to intestinal injury, such as volvulus and intussusception [29]. Similarly, in human medicine, the so-called intestinal malrotation is a congenital anomaly resulting from incomplete rotation and fixation of the intestine during embryological and fetal development, which may predispose to midgut volvulus and lead to duodenal obstruction and strangulation of the circulation in the superior mesenteric vessels [30]. In humans and mice, *MAP2K2* is known to be highly expressed in the colon and duodenum [31,32]. However, mutations in *MAP2K2* have so far not been associated with enteric congenital defects.

Furthermore, the deleterious nature of the variant and the conservation of the affected arginine amino acid residue of *MAP2K2* at position 179 in protein kinase domain also suggest that this variant is most likely pathogenic. Indeed, mutations of *MAP2K2* affect the negative regulatory region or the core catalytic domain of the kinases, leading to increased kinase activity and gain-of-function effect on the RAS/MAPK pathway [28,33,34].

## 5. Conclusions

The investigation of this case enabled a pathological and molecular genetic study that, for the first time, allowed the diagnosis of a dominantly inherited skeletal-cardio-enteric dysplasia in a calf associated with a *MAP2K2* missense variant. Thus, we hereby present the first large animal model for similar human diseases. Furthermore, this example highlights the usefulness of genetically precise diagnosis for understanding sporadic cases of congenital disorders caused by de novo mutations, and the need for continuous monitoring of genetic lethal disorders in cattle breeding. Additionally, novel discoveries in large animals such as cattle are useful as biomedical models for human diseases.

## Data Availability

The whole-genome data of the affected calf (sample ID PCS1780) is freely available at the European Nucleotide Archive (ENA) under sample accession number SAMEA7690195.

## Supplementary Materials

The following are available online at https://www.mdpi.com/article/10.3390/ani11071931/s1, Table S1: EBI Accession numbers of all publicly available genome sequences. We compared the genotypes of the calf with 598 cattle genomes of various breeds that had been sequenced in the course of other ongoing studies and that were publicly available, Table S2: List of the remaining variants after the comparison to the global control cohort of 4110 genomes of other breeds (1000 Bull Genomes Project run 8; www.1000bullgenomes.com; acceded on 30 April 2021) and after IGV visual inspections, revealing 63 protein-changing variants with a predicted moderate or high impact only present in the affected calf. Figure S1: Newborn Romagnola healthy calf. Figure S2: Radiographic image of the thoraco-lumbar region of the affected calf.
