# Supplementary figures and images for: A Heterozygous Missense Variant in MAP2K2 in a Stillborn Romagnola Calf with Skeletal-Cardio-Enteric Dysplasia
Source: Animals (Basel). 2021 Jun 29;11(7):1931. doi: 10.3390/ani11071931 (PMC8300254; doi:10.3390/ani11071931)

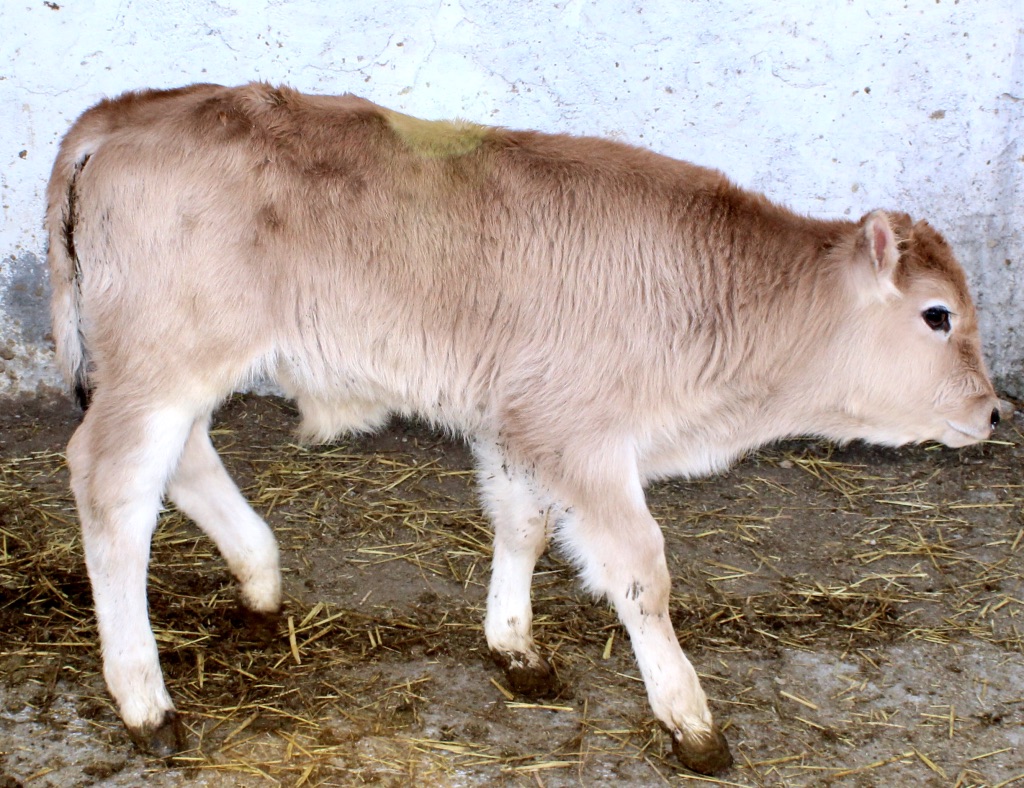

Supplement: Supplementary file 1 [file animals-11-01931-s001.zip › Figure_S1.jpeg]

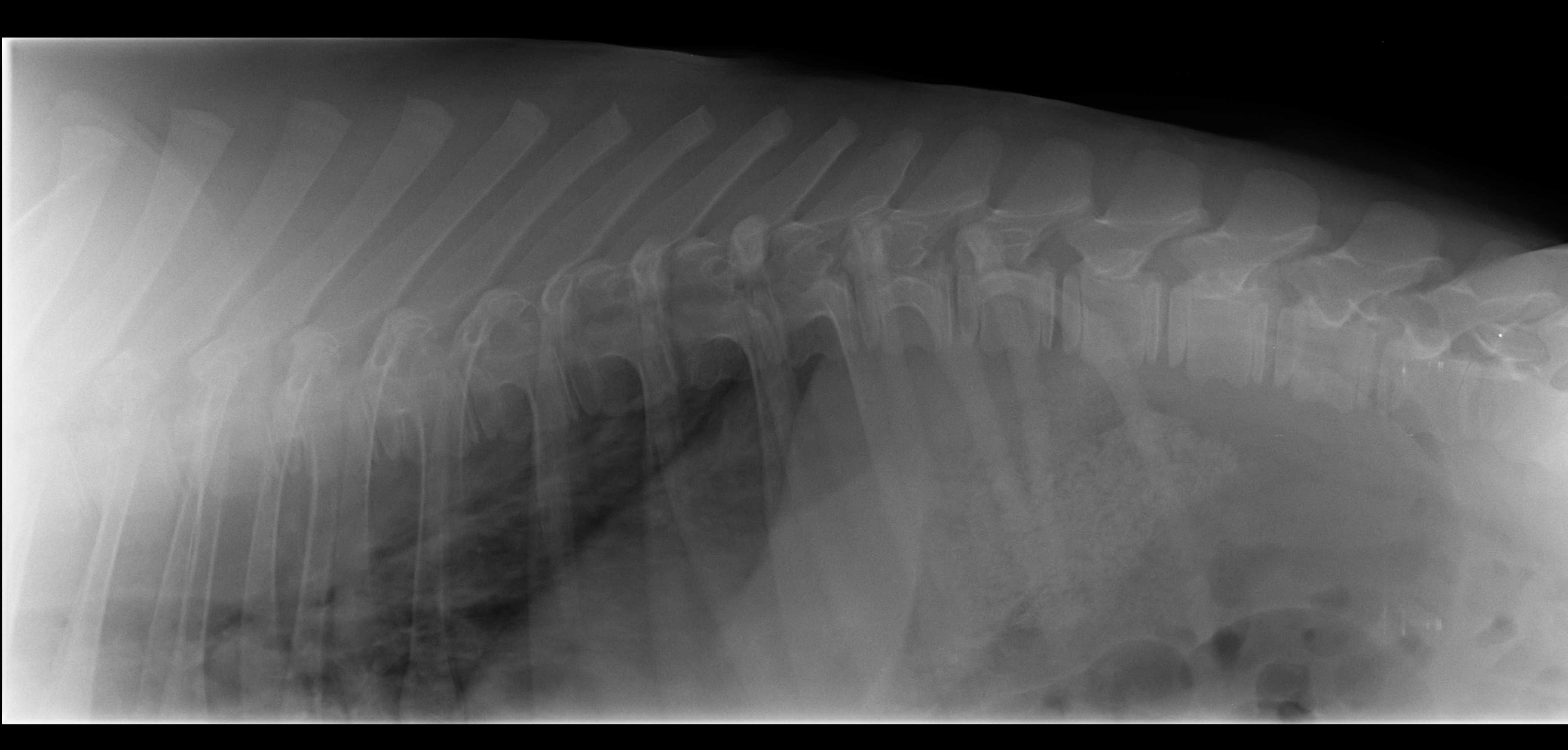

Supplement: Supplementary file 1 [file animals-11-01931-s001.zip › Figure_S2.jpg]
